# Bacteria on the foundational kelp in kelp forest ecosystems: Insights from culturing, whole genome sequencing and metabolic assays

**DOI:** 10.1111/1758-2229.13270

**Published:** 2024-05-22

**Authors:** Isaac T. Younker, Nichos Molnar, Kaylie Scorza, Roo Weed, Samuel H. Light, Catherine A. Pfister

**Affiliations:** ^1^ Committee on Microbiology The University of Chicago Chicago Illinois USA; ^2^ The College The University of Chicago Chicago Illinois USA; ^3^ The Graduate Program in Biophysical Sciences The University of Chicago Chicago Illinois USA; ^4^ Department of Microbiology The University of Chicago Chicago Illinois USA; ^5^ Department of Ecology and Evolution The University of Chicago Chicago Illinois USA

## Abstract

In coastal marine ecosystems, kelp forests serve as a vital habitat for numerous species and significantly influence local nutrient cycles. Bull kelp, or *Nereocystis luetkeana*, is a foundational species in the iconic kelp forests of the northeast Pacific Ocean and harbours a complex microbial community with potential implications for kelp health. Here, we report the isolation and functional characterisation of 16 *Nereocystis*‐associated bacterial species, comprising 13 *Gammaproteobacteria*, 2 *Flavobacteriia* and 1 *Actinomycetia*. Genome analyses of these isolates highlight metabolisms potentially beneficial to the host, such as B vitamin synthesis and nitrogen retention. Assays revealed that kelp‐associated bacteria thrive on amino acids found in high concentrations in the ocean and in the kelp (glutamine and asparagine), generating ammonium that may facilitate host nitrogen acquisition. Multiple isolates have genes indicative of interactions with key elemental cycles in the ocean, including carbon, nitrogen and sulphur. We thus report a collection of kelp‐associated microbial isolates that provide functional insight for the future study of kelp–microbe interactions.

## Graphical abstract

We isolated 16 distinct bacterial taxa across three phyla from the surface of the bull kelp *Nereocystis luetkeana* at three sites in Washington State. Whole genome sequencing of the 16 isolates suggested a range of metabolisms that may benefit kelp, and laboratory assays showed ammonification that generated ammonium, a potential source of dissolved inorganic nitrogen for the host. Photo credit: Steve Lonhart, Tatoosh Island, Washington, USA.

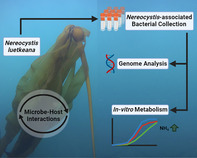

## INTRODUCTION

A growing appreciation of host–microbe (the holobiont) interactions has altered our view on how species evolve (Rudman et al., 2019) and contend with environmental stress (Dittami et al., 2016; Van Oppen & Medina, 2020). In coastal marine ecosystems, macroalgae and seagrasses are increasingly recognised as hosts of microbiomes that include nitrogen‐fixing taxa (Cardini et al., 2018; Mohr et al., 2021), carbon‐degrading taxa (Weigel et al., 2022), and impact overall host health either positively or negatively (Li et al., 2022). Factors provided by associated microbes are important for macrophyte health, as demonstrated by developmental abnormalities in axenic seaweed cultures (Croft et al., 2005; Ghaderiardakani et al., 2017). Despite this increasing evidence that macrophytes host important microbes, much remains unknown about the molecular basis underlying host–microbe interactions.

Macrophytes often play foundational roles within coastal marine ecosystems. For example, kelp forests provide habitat for hundreds of species (Bodkin, 1986), are hotspots for biogeochemical cycling, including carbon fixation (Koweek et al., 2017; Pfister et al., 2019), and contribute to the coastal food webs in the form of kelp detritus (Duggins et al., 1989). Furthermore, canopy‐forming kelp have been shown to host diverse assemblages of microbes (Minich et al., 2018; Osborne et al., 2023; Weigel et al., 2022; Weigel & Pfister, 2019). One such example is the bull kelp *Nereocystis luetkeana* (henceforth, *Nereocystis*), a canopy‐forming species in the northeast Pacific from Alaska to California (Druehl, 1970). *Nereocystis* is highly productive, with a biomass production of up to 2.35 kg/m^2^ (North, 1994; Weigel & Pfister, 2020), and lengths of up to 20 m. *Nereocystis* spans a strong environmental gradient in Washington State, from the outer coastal areas in the western part of the state where kelp populations have been persistent (Pfister et al., 2018) to the southern end of Puget Sound, where populations have declined as much as 63% (Berry et al., 2021), likely due to increased seawater temperatures in Puget Sound (Greene et al., 2015). Perhaps relating to these environmental differences, *Nereocystis* microbial communities across this geographic extent differ (Weigel & Pfister, 2019). The southernmost population in Puget Sound exhibits a comparatively depauperate microbiome compared to the robust outer coast population at Tatoosh Island, where imaging indicated 10^5^–10^7^ bacterial cells/cm^2^ on the blade region of *Nereocystis* (Ramírez‐Puebla et al., 2022).

Despite the growing knowledge of microbes associated with kelp (King et al., 2022, 2023), relatively little is known about the contribution of these microbes to the health of their hosts. Metabolic analyses, using metagenomics or 16S amplicon sequencing, have grouped the metabolisms of multiple taxa into broad categories (Lin et al., 2018; Miranda et al., 2022; Selvarajan et al., 2019; Weigel et al., 2022). Searches for metabolic genes in metagenomes and the analysis of individual metagenome‐assembled genomes identified several metabolisms (including vitamin production, carbohydrate use, and nitrogen metabolism) that may be important for kelp health (Miranda et al., 2022; Weigel et al., 2022).

Limited nitrogen accessibility can negatively impact macrophyte growth in the coastal ocean. While most marine macrophytes rely on dissolved inorganic nitrogen (DIN) for their nitrogen needs, bacteria can use nitrogen in many forms, including dissolved organic nitrogen (DON). DON as a bacterial nitrogen source is likely important to oceanic nitrogen cycling (Berman & Bronk, 2003; Bronk et al., 2007). One bacterial metabolism that can be beneficial to the host is the ammonification of amino acids. Bacteria cleave the carbon‐nitrogen bonds of amino acids, potentially making DIN available as ammonium, a process that has been demonstrated in seagrasses using stable isotopes and elemental imaging (Tarquinio et al., 2018) and is hypothesised to occur in the *Nereocystis* microbiome as well (Hochroth and Pfister, 2024). Carbon resources that microbes could use include a diversity of polysaccharides either from the kelp (Thomas et al., 2021) or the abundant chitin from surrounding seawater (Larsbrink et al., 2016). Additionally, the high densities of microbes on hosts (Ramírez‐Puebla et al., 2022) might lead to competitive or allelopathic interactions among bacteria, including the membrane attack perforin proteins (MACPF, McEneany et al., 2018).

Emerging knowledge about microbes in coastal marine areas includes descriptions of their ability to produce biodegradable plastic (Moriya et al., 2020), provision vitamins (Degnan et al., 2014) and produce secondary metabolites medically important to humans (Modolon et al., 2020; Penesyan et al., 2009). The multiplicity of functions that may have implications for human and ocean health motivates increased investigation into the metabolisms of marine bacteria.

Here, we describe microbes isolated from the surface of *Nereocystis*, with an emphasis on the metabolic capabilities of bacteria that live on its surface. To enhance our understanding of the identity and possible functions of individual microbial taxa, we isolated bacteria from the surface of *Nereocystis* blades across three sites. Following isolation in culture, we sequenced the whole genome of each isolate (whole genome sequencing [WGS]). We further designed several metabolic assays—including quantifications of growth advantage and ammonium production in the presence of amino acids—to investigate the potential of our isolates to contribute to carbon and nitrogen cycling for both the host and the surrounding environment. We report bacterial metabolisms that may benefit the kelp host as well as metabolisms that might benefit bacteria that are host‐associated.

## EXPERIMENTAL PROCEDURES

### Kelp blade sample collection

**FIGURE 1:**
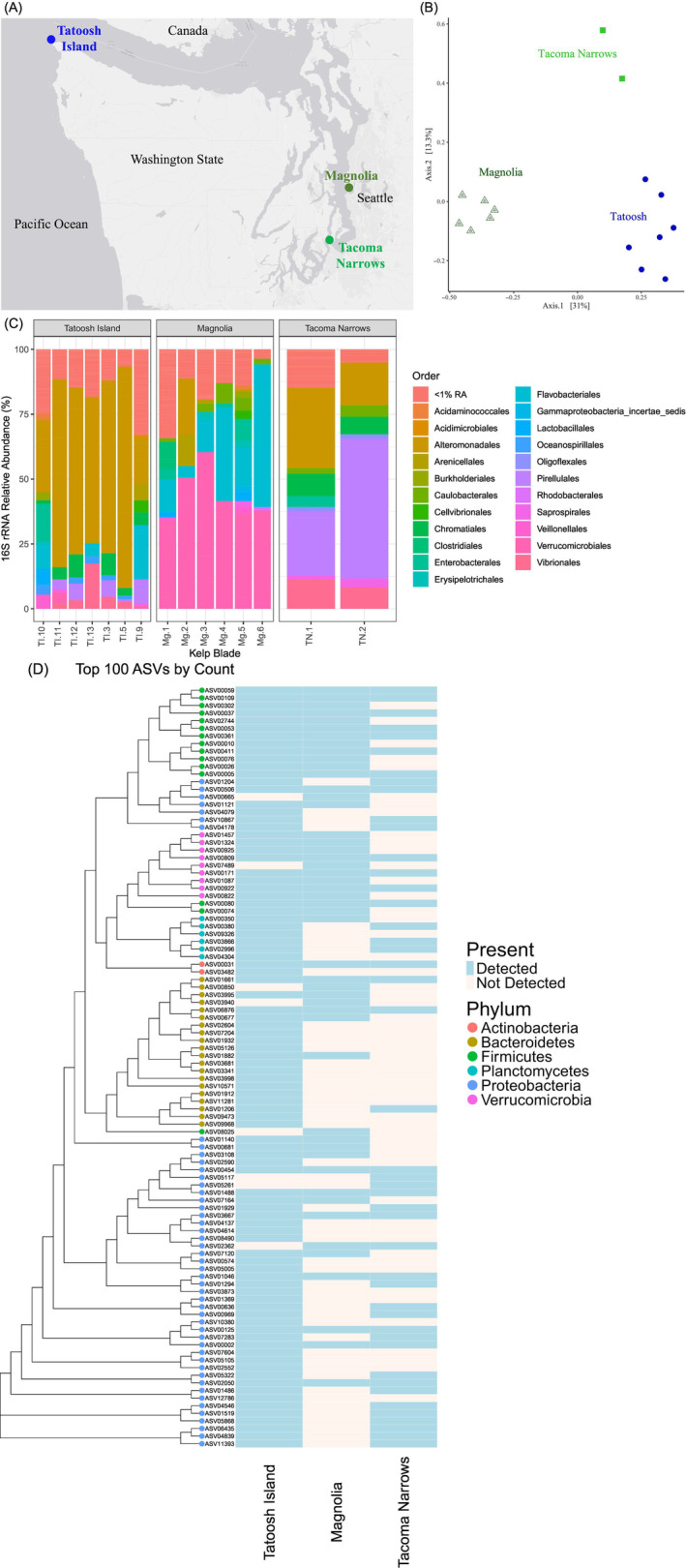
(A) Map of the three sampling areas for the *Nereocystis luetkeana* microbiome in Washington State. (B) The distinctness of amplicon sequence variants (ASVs) from 16S rRNA amplicon sequences from three collection sites portrayed with principal coordinate analysis (PCoA) and based on Bray distance matrices. All ASVs are shown, except for those that were only detected once across all 15 samples. The clusters differed significantly (permanova, *F*_2,14_ = 4.452, *p* = 0.001, df = 14). (C) The representation of ASV diversity by order on individual kelp blades prior to culture efforts. ASVs are based on 16S rRNA amplicon sequencing of kelp homogenates. All ASVs are provided in Table S2. (D) A heat map of the relative abundance of the top 100 ASVs from each of the three sites sampled. The observed diversity per sample was 129.0 ASVs for Tatoosh (*n* = 7), and 122.3 and 74.0 for Magnolia (*n* = 6) and Tacoma Narrows (*n* = 2), respectively.

We assessed kelp microbial communities across three distinct sites in Washington State. Individual *Nereocystis* blades were collected between September and October 2021 from the north side of Tatoosh Island (*n* = 7) (48.393689, −124.733820) on 6 September 2021 and from Tacoma Narrows (47.296389, −122.531944) and Magnolia (47.630833, −122.398333) (*n* = 6 each) on 19 September 2021 and 4 October 2021, respectively (Figure 1A). Samples were placed in individual plastic bags, kept chilled (~12°C), and transported to the University of Chicago, where they were censused within 48 h. Following aseptic technique, a ~2 cm^2^ section was removed from each *Nereocystis* blade and cut into small pieces, placed in Dulbecco's phosphate‐buffered saline without magnesium/calcium (DPBS; 160 g/L NaCl, 4 g/L KCl, 4 g/L KH_2_PO_4_, 23 g/L Na_2_HPO_4_, pH 7.4) and vortexed for 1 min to liberate bacteria from the surface of the kelp. Resulting mixed bacterial stocks were used for 16S rRNA amplicon sequencing and stored as glycerol stocks at −80°C for subsequent culturing.

### Bacterial isolation and culturing

Each mixed bacterial stock was serially diluted in DPBS and 100 μL spread on agar plates made with ‘marine LB’ (MLB; Luria‐Bertani broth supplemented to a 3% final concentration NaCl) + 10 mM NH_4_Cl or 10% MLB plates. Unique colonies were selected and restruck (passaged) on agar plates to visual purity (~3 passages); individual representative colonies of each isolate were saved as stocks in 20% glycerol‐DPBS at −80°C. All bacteria were grown in an environmental chamber maintained at a 14:10 light:dark cycle ranging from 12 to 14°C and under 50 μM of photosynthetically active radiation, conditions that mimic approximately 1–2 m depth in the coastal northeast Pacific Ocean, where this kelp species is indigenous (Pfister et al., 2019).

We used several compositions of growth media to maximise the number of bacterial taxa we could isolate from the kelp surface. We used MLB, 10% MLB and M13 (DSMZ #607) media, all with and without liquid from a puree of *Nereocystis*. Kelp puree and extract were made from *Nereocystis* from Tatoosh Island, WA. Kelp (100 g) was pureed with a hand blender in 200 mL of sterile‐filtered seawater and immediately frozen. This puree was thawed, centrifuged at 2000 rpm for 1 min and the supernatant forced through a 0.20 μm sterile syringe filter (Corning). This concentrate was added to each of the three media types at a 0.8% final concentration. Each media condition was directly inoculated using the same kelp homogenate glycerol stock from Tatoosh (TI #9), grown with aeration for 72 h and then back‐diluted 1:100 in fresh media (one passage). Cultures were passaged four times, at which point an aliquot was saved as a glycerol stock for future isolation efforts and the remaining culture was prepared for 16S rRNA amplicon sequencing. We also used 16S rRNA amplicon sequencing on the four aliquots of our kelp extract samples to determine if there were bacteria present. When we analysed these samples with the 16S rRNA amplicon pipeline described below, we did not filter out any singletons to enhance our detection of contamination.

### 16S amplicon sequencing

DNA from kelp homogenates or cultured growth media was extracted using the QIAamp PowerFecal Pro DNA kit (Qiagen). Prior to extraction, samples were subjected to mechanical disruption using the bead beating method, where samples were suspended in a bead tube (Qiagen) containing a lysis buffer and loaded on a bead mill homogeniser (Fisherbrand). Samples were then centrifuged, and the supernatant was resuspended in a reagent that effectively removed inhibitors. DNA was then purified using a spin column filter membrane and quantified using Qubit.

DNA was amplified, sequenced and amplicon sequence variants (ASVs) were identified by the University of Chicago Duchossois Family Institute Microbial Metagenomics Facility (DFIMMF) at The University of Chicago. The V4–V5 region within the 16S ribosomal RNA (rRNA) gene was amplified using universal bacterial primers—563F (5′‐nnnnnnnn‐NNNNNNNNNNNN‐AYTGGGYDTAAA‐GNG‐3′) and 926R (5′‐nnnnnnnn‐NNNNNNNNNNNN‐CCGTCAATTYHT‐TTRAGT‐3′), where ‘N’ represents the barcodes and ‘n’ is an additional nucleotide added to offset primer sequencing. Polymerase chain reaction conditions included initial denaturation at 94°C for 3 min followed by denaturation, annealing and extension at 94°C/15 s, 51°C/30 s and 72°C/1 min. Final extension was performed for 5 min. For initial sequencing of the kelp homogenates, 35 cycles were run to maximise amplification, while 27 cycles were run for cultures that were enriched (Table S3). Approximately ~412 bp region amplicons were then purified using a spin column‐based method (Minelute, Qiagen), quantified and pooled at equimolar concentrations. Dual index adapters were ligated onto pooled amplicons, and sequences were generated from the Illumina MiSeq platform using the QIASeq 1‐step amplicon kit (Qiagen) for generating libraries and using 2 × 250 Paired End reads with 5000–10,000 reads per sample.

We used dada2 (v1.18.0) as our default pipeline for processing MiSeq 16S ribosomal RNA (rRNA) reads with minor modifications in R (v4.0.3). Reads were first trimmed at 190 bp for both forward and reverse reads to remove low quality nucleotides, and chimaeras were detected and removed using the default consensus method in the dada2 pipeline. Then, ASVs with length between 320 and 365 bp were kept and deemed as high‐quality ASVs. Taxonomy of the resultant ASVs was assigned to the genus level using the Ribosomal Database Project classifier (v2.13) with a minimum bootstrap confidence score of 80. Species‐level classification used BLASTn (v2.13.0) and the refseq_rna database.

### Alpha‐ and beta‐diversity analyses

We estimated alpha‐ and beta‐diversity using R and phyloseq (R version 2022.12.0+353), after pruning only ASVs that occurred once across all samples and removing sequences that were chloroplasts.

### Whole genome sequencing

Single colonies of pure culture were used to inoculate a broth culture of 4 mL of MLB + NH4. After 4 days of growth at 13.5°C on a shaker, 3 mL of each culture was pelleted and washed once in DPBS. Supernatant was removed and the bacterial pellet frozen at −80°C prior to submission for WGS to the DFIMMF. All extraction, sequencing, and assembly were done by the DFIMMF. Briefly, DNA for WGS was extracted with QIAamp PowerFecal Pro DNA Kit. Libraries compatible with Illumina were generated with QIAseq FX Library Kit. Sequences were generated on Illumina NextSeq with 2 × 150 paired end reads with 1,000,000 to 3,000,000 reads per isolate. Assembly was done with SPADES and annotation with prokka.

### Metabolic pathway analysis

WGS contigs.fasta files were analysed with anvi'o (v 7.1, Eren et al., 2021), first using anvi‐gen‐contigs‐database. We used anvi‐run‐kegg‐kofams to calculate the KEGG hits, annotating KEGG orthologues (KOs) as present with an e‐value threshold of 1e−05 and a bitscore fraction of 0.5, the default values in anvi'o, and based on pre‐defined bitscore thresholds (an ‘adaptive threshold,’ Aramaki et al., 2020) for all KOs. We used anvi‐estimate‐metabolism to identify metabolic pathways in each genome. Metabolisms were described as modules and a module was considered present if 75% of the KEGG genes (KOs) necessary to complete the metabolic pathway were identified, the default result in the anvi'o workflow (Watson et al., 2023). We also examined clusters of orthologous genes and Pfam annotations. We analysed metabolic differences among bacterial taxa using the matrix of KEGG hits and metabolic modules for each taxon.

We quantified metabolisms with high potential significance in host–microbe interactions, including microbial metabolisms that increase host access to nitrogen, such as dissimilatory nitrate reduction (module M00530) and nitrogen fixation (KO2588). As amino acid metabolisms such as ammonification could increase ammonium locally, we searched for Enzyme Commission (EC) numbers within each genome that would indicate enzymes that act on CH‐NH2 bonds (EC:1.4.*), enzymes that act on carbon‐nitrogen bonds other than peptides (EC:3.5.*) or ammonium lyases (ED:4.3.1*), where asterisk indicates any subset of these classifications; all corresponding KO numbers are in Table S1. Additional microbial metabolisms that could benefit the host include B vitamin synthesis, including vitamin B_1_ (thiamine, M00127), B_2_ (riboflavin, M00125), B_6_ (pyridoxine, M00124, M00916), B_7_ (biotin, M00123) and B_12_ (cobalamin, M00122). We also searched for genes that could aid microbes in responding to reactive oxygen stress from the host, including reactive oxygen species (ROS) response genes *oxyR*, *soxR* and *katG*.

### Bacterial growth assays

We tested the effect of different amino acids on the growth rates of each isolate. Starter cultures were grown for ~36 h in M13 media; 3 mL of media was then pelleted and washed three times in 1 mL of DPBS. We supplemented 6 mL of experimental cultures in M13 media with an individual amino acid at a concentration of 10 mM, except for glutamate, which was assayed at 5 mM in some experiments. All were inoculated at a final OD600 = 0.001 and grown with aeration at 14.5°C. At each timepoint, 200 μL of each culture was transferred to a 96‐well plate (GreinerBioOne) in duplicate to estimate growth rates using optical density measurements (Biotek Cytation 6 microplate reader). Additionally, a 1 mL aliquot from each culture was taken at approximately late log and stationary phase, centrifuged at 20,200 × *g* for 5 min at room temperature, the supernatant transferred to a clean 1.5 mL microtube, and stored at −20°C until ammonium quantification. Controls were grown in M13 media only. We assessed growth on amino acids that have a single nitrogen atom (alanine, serine, aspartate and glutamate) as well as amino acids with two nitrogen atoms (asparagine and glutamine). Bacterial growth on agmatine, a 3‐amine derivative of arginine, was also assayed.

### Ammonium quantification

Bacterial production of ammonium was quantified in each well with a fluorometric assay (Holmes et al., 1999). Standard curves were made with an ammonium standard that was serially diluted (5‐ to 4000‐fold). Dilutions were then mixed (250 μL:1 mL) with either sodium tetraborate buffer (40 g/L) or working reagent (sodium tetraborate; 40 g/L, sodium sulphite; 40 mg/L, and o‐phthalaldehyde in ethanol; 1 mg/mL), inverted to mix and allowed to react for 2 h in the dark. After development, each reaction was measured in triplicate in a black 96‐well microplate. Fluorescence was measured using a Cytek 5 with 350 ± 9 nm excitation and 422 ± 20 nm emission for 25 flashes. We compared ammonium production on amino acids with differing numbers of nitrogen atoms using a linear mixed effects model with a bacterial strain as a random effect because not all amino acids were tested across all isolates (‘nlme’ in R). All statistical analyses were done with RStudio Version 2022.12.0, Build 353.

## RESULTS

### Kelp microbial communities are diverse

We discovered the greatest taxonomic diversity at Tatoosh Island with an average of 129.0 ASVs/sample, compared to a mean of 122.3 observed ASVs/sample at Magnolia, and 74.0 at the Tacoma Narrows (Table S2; Figure 1). The relative ranking of diversity did not change when we rarified the data (80.1, 73.8 and 52.5 ASVs/sample, respectively). Beta‐diversity differed by site (Figure 1B, permanova, *F* = 4.452, *p* = 0.001, df = 14), where *Proteobacteria* dominated at Tatoosh Island and Tacoma Narrows, and *Verrucomicrobia* was the most abundant phylum at Magnolia (Table S2). Differences at the order level were apparent across locales with an increased relative abundance of *Alteromonadales* at Tatoosh and increased *Clostridiales* at Magnolia (Figure 1C).

Despite taxonomic differences across the sites, many of the most abundant ASVs were present at multiple sites (Figure 1D). Notably, a subset of ASVs present at Tatoosh Island was not detected in the Magnolia and Tacoma Narrows samples (Figures 1D and S1). These results show that the Puget Sound samples broadly possess similar, but less diverse, microbial communities and specifically lack a subset of taxa present at Tatoosh Island.

### Kelp nutrients promote growth of specific kelp‐associated bacteria

To facilitate the isolation of *Nereocystis*‐associated microbes and identify potential kelp–microbe interactions, we tested the effect of kelp extract on microbial growth from our *Nereocystis* isolates. In both media tested, we observed that supplementing with kelp extract increased the number of taxa detected by 16S amplicon sequencing (Figure S2). We detected three bacterial strains that were unique to the addition of kelp extract to agar media, including two from the phylum *Bacteroidota* (the *Flavobacteriia* *Lishizhenia* and *Flavobacterium*) and one from the phylum *Gammaproteobacteria* in the *Halomonadaceae* (*Cobetia*), suggesting that kelp extract promoted the growth of novel taxa. The 10% MLB agar did not show an increase in taxa discovery with kelp extract.

### *Establishing a* Nereocystis*‐associated bacterial species isolate collection*

**FIGURE 2:**
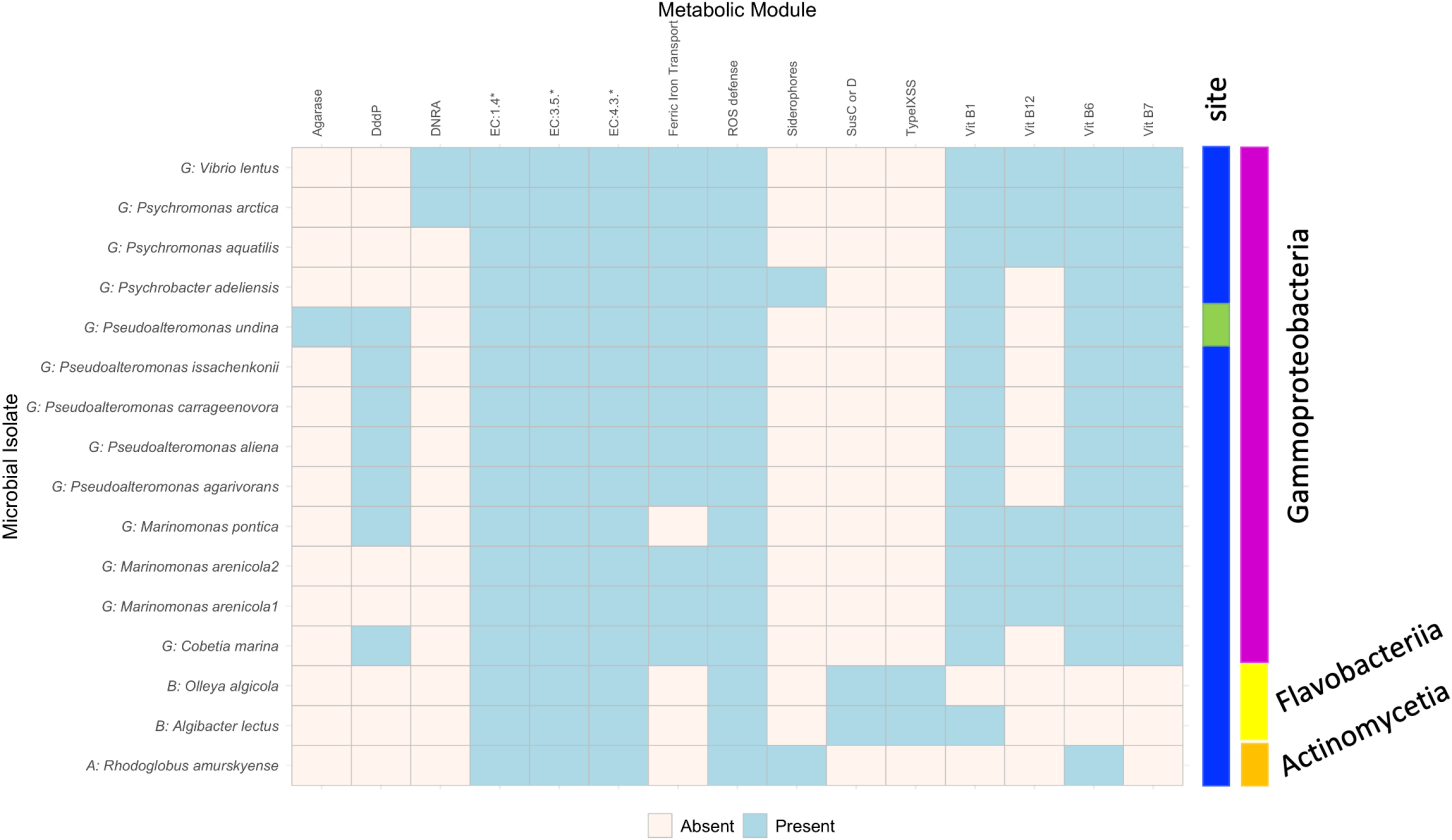
Key metabolisms from the microbial taxa that have been cultured and sequenced from the surface of *Nereocystis*. Except for *Pseudoalteromonas undina*, which was cultured from Tacoma Narrows (green), all taxa were cultured from Tatoosh Island *Nereocystis* (blue). Metabolisms that could be important to host–microbe interactions are shown as either present or absent based on module completeness, the presence of a KEGG orthologue gene or a protein (Pfam). Full metabolic results are shown in Tables S4 and S5.

Twelve species within *Gammaproteobacteria* were identified from single colonies of *Nereocystis* mixed bacterial samples from Tatoosh, Magnolia and Tacoma Narrows (Figure 2; Table S3). Pre‐passaging in kelp‐supplemented liquid media led to the discovery and isolation of four additional taxa, including two *Flavobacteriia* genera in the *Bacteroidota* phylum (*Algibacter* and *Olleya*) and a *Actinomycetia* (*Rhodoglobus*), as well as another strain of *Marinomonas*. Consistent with the reduced microbial diversity in Puget Sound, we only isolated a single unique species (*Pseudoalteromonas undina*) and no unique genera from the Tacoma Narrows and Magnolia samples. Indeed, 10 isolates from Tacoma Narrows and 6 isolates from Magnolia were redundant with Tatoosh isolates. Visual inspection of our cultures also indicated consistently fewer culturable cells for Tacoma Narrows and Magnolia compared with Tatoosh (Figure S3). From our isolates, we selected representative strains from 16 assigned species to establish a collection of *Nereocystis*‐associated bacterial isolates for which we performed WGS; genome features are shown in Table S3.

### Kelp‐associated bacteria have metabolic capabilities complementary to the host

Bioinformatic analysis of the metabolic potential of our isolates identified 24 metabolic modules encoding highly conserved functionalities (core carbohydrate, amino acid, fatty acid and nucleotide metabolisms, etc.) in all 16 genomes (Figure S4; Table S4). We further identified a number of genes present in a subset of isolates with potential biological and/or ecological implications elaborated below (Figure 2; Table S3).

#### Vitamin biosynthesis

Synthesising and provisioning vitamins represent key mechanisms of host–microbe interactions. A majority of our isolates, including all of the *Gammaproteobacteria*, possess biosynthetic pathways for the production of vitamins B_1_ (thiamin), B_6_ (pyridoxal) and B_7_ (biotin) (Figure 2). Five *Gammaproteobacteria* isolates also have the vitamin B_12_ (cobalamin) biosynthetic pathway. Notably, the *Flavobacteria* and *Actinobacteria* isolates in our collection are predicted to be auxotrophic for multiple vitamins and may depend upon other members of the microbial community for essential micronutrients.

#### Ammonium production

Although nitrogen utilisation is important in the ocean, we did not detect nitrogen fixation, denitrification or assimilatory nitrate reduction genes in any of our strains. *Psychromonas arctica* and *Vibrio lentus* isolates encoded dissimilatory nitrate reduction genes. Amino acid deaminases, a potential source of amino acid‐derived ammonium, were present across all taxa and in all EC categories that describe these enzymes (Figure 2; Tables S4 and S5), suggesting that microbial isolates within our collection may generate ammonium from nitrate and amino acids.

#### Others

We identified additional genes that could be important for host–microbe interactions. Genes for ferric iron transport (*fhu* genes) were present in 12 of the 13 *Gammaproteobacteria* strains (Figure 2). Interactions with sulphur are predicted to occur in seven strains from the genera *Pseudoalteromonas*, *Marinomonas* and *Cobetia* that have the gene Dddp to catalyse conversion of the kelp metabolite dimethylsulfoniopropionate (DMSP) into dimethyl sulphide (DMS) (Figure 2). However, no bacterial isolate appears to produce DMSP, based on the lack of an additional DMSP synthesis gene, *dsyB* (Table S3). All bacterial isolates have some genes that could be responsive to ROS, including *oxyR*, *soxR*, *katG*, though *Rhodoglobus amurskyense*, *Psychrobacter adeliensis* and *Cobetia marina* had the fewest matches (Figure 2). Only *P. undina*, the sole species cultured from the Tacoma Narrows site, had agarase genes that could enable degradation of the kelp polysaccharide agar, potentially either inducing host damage or enabling microbial utilisation of dead kelp. Finally, *Flavobacteria* strains had *susC* and *susD*‐like transporter genes, which could indicate oligosaccharide uptake capabilities, and Type IX secretory systems.

### Kelp‐associated bacteria metabolise amino acids and produce ammonium

**FIGURE 3:**
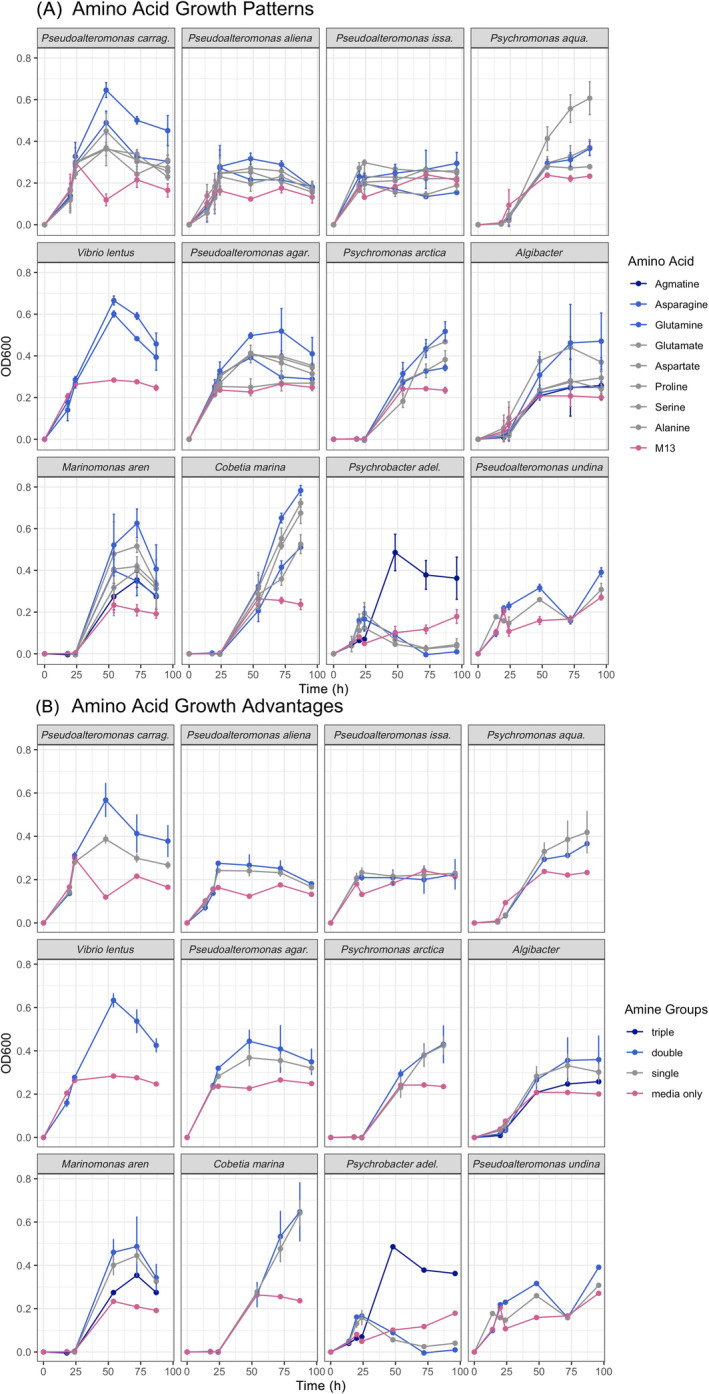
Growth of 12 isolates when different amino acids were added to M13 media in liquid culture. Growth curves are colour‐coded by the number of nitrogen atoms per amino acid. In (A), growth on every amino acid, quantified with OD600, (B) has amino acids grouped into one, two or three nitrogen atoms per amino acid, or the media only. Agmatine was the only amino acid with three nitrogen atoms. At the 72‐h time point, bacterial growth differed based on the number of nitrogen atoms (*F*_3,30_ = 5.93, *p* = 0.003, Table S6) and across all strains. Through time, growth on amino acids with two nitrogen atoms was greater across all strains (repeated measures ANOVA, amine group *F*_1,282_ = 5.56, *p* = 0.019, and strain *F*_11,282_ = 9.46, *p* < 0.001).

All bacterial isolates grew better with the addition of an amino acid, except *P. adelensis* (Figure 3A). At the 72 h time point, bacterial growth was positively correlated with the number of nitrogen atoms contained within each amino acid (*F*_3,30_ = 5.93, *p* = 0.003, Figure 3B); the mean optical density of isolates on M13 media supplemented with amino acids with two nitrogen atoms showed a 67% growth increase relative to unsupplemented M13 media. When we compared amino acids with a single nitrogen atom to those with two nitrogen atoms and included all timepoints, repeated measures analysis of variance (ANOVA) indicated that growth on glutamine and asparagine (amino acids with two nitrogen atoms) was greater than growth on amino acids with a single nitrogen atom, though the growth trajectories of different strains varied (Table S6).

**FIGURE 4:**
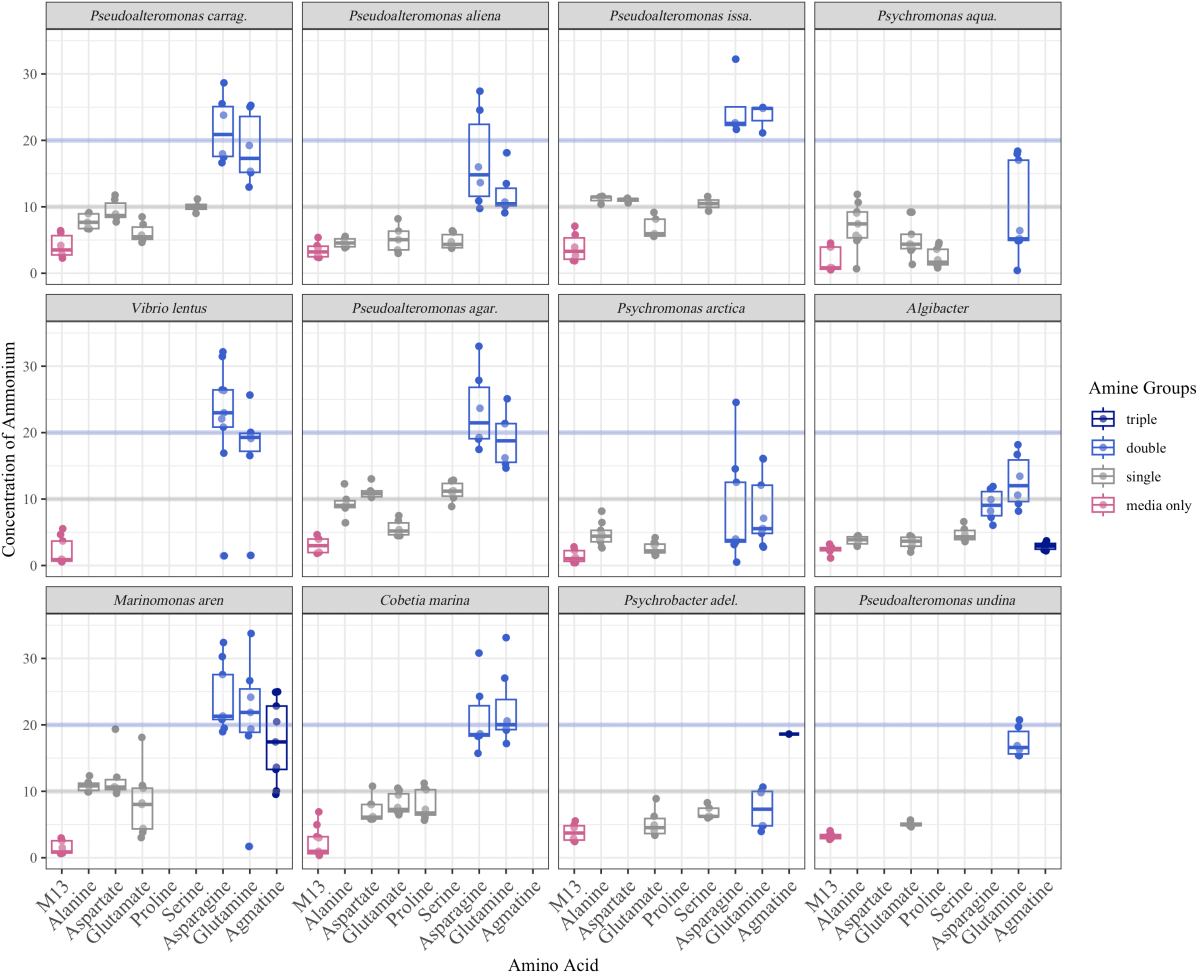
Ammonium production in (mM) by each of 12 *Nereocystis*‐associated isolates grown on amino acids with differing number of nitrogen atoms. Amino acids are colour‐coded based on the number of nitrogen atoms. Ammonium production was greatest when isolates were grown on amino acids with two nitrogen atoms (linear mixed effects model, *p* < 0.001, Table S6). The horizontal blue line designates concentrations if amino acids with two nitrogen atoms are completely converted to ammonium; the grey line is for amino acids with a single nitrogen atom. An asterisk indicates instances where glutamate was used at 5 mM rather than 10 mM.

When bacteria utilise amino acids as a carbon or energy source, they typically generate ammonium as a reaction product. Most isolates produced detectable ammonium concentrations when grown on M13 media only or media amended with amino acids (Figure 4). Consistent with strains possessing efficient amino acid metabolisms, we observed ammonium levels (1) correlated with the number of nitrogen atoms present in provisioned amino acids (1‐ and 2‐containing amino acids produced an average of 6.79 and 18.26 mM ammonium, respectively) and (2) approached the high (mM) concentrations expected for complete amino acid consumption (Figure 4). These results demonstrate that kelp‐associated bacterial isolates metabolise amino acids and could provide an ecologically relevant source of ammonium.

## DISCUSSION

### Kelp‐associated bacterial metabolisms

Association with seaweeds presents both costs and opportunities for bacteria. Seaweeds have been demonstrated to represent a strongly hyperoxic surface (Irwin & Davenport, 2002), with the potential to produce several ROS (Hansel & Diaz, 2021). Seaweeds can release hydrogen peroxide (H_2_O_2_), hydroxyl radicals (OH•) or superoxide ions (O_2_^−^•) (Hansel & Diaz, 2021; Weinberger, 2007), and kelp specifically have been shown to release large quantities of H_2_O_2_ upon application of a cell wall degradation product associated with pathogens (Küpper et al., 2001). The mechanisms that allow seaweeds and potentially mutualistic bacteria to withstand this strong ROS response remain unknown. Defence against ROS lead to a well‐documented dependency in *katG* in the abundant oceanic cyanobacteria *Prochlorococcus* and *Synecococcus* that were the inspiration for the Black Queen Hypothesis (Morris et al., 2012), where genome reduction in *Prochlorococcus* resulted in reliance on neighbours for protection from ROS. Microbes have responses to ROS that include the enzymes *oxyR*, *perR* and *soxR* (Imlay, 2015; Jo et al., 2015), among others (Johnson & Hug, 2019) and we found ROS enzymes in all microbial isolates (Figure 2, Table S3).

Seaweeds are also known to produce DMSP (Alstyne et al. 2015), an abundant organosulphur compound in the ocean. Although DMSP has been shown to deter some marine bacteria (Saha et al. 2012), seven bacterial isolates in our study had the lyase enzyme (Dddp) to potentially catalyse the cleavage of DMSP into DMS (Figure 2). However, none of our bacterial isolates appear to produce DMSP, based on the lack of a*dsyB* gene (Table S4), suggesting that microbes are responsive to host function. Enhancing our strictly genomic study with the analysis of the prevalence and taxonomic distribution of DMSP‐cleaving genes, including whether expression of these genes elicits a response in seaweed DMSP production (Van Alstyne et al., 2023), would contribute to our understanding of whether seaweed‐associated microbes convert DMSP into DMS, an ‘anti‐greenhouse gas’ (Todd et al., 2007).

Seaweeds, including the kelp *Nereocystis* produce an abundance of dissolved organic compounds (Paine et al., 2021; Weigel & Pfister, 2020) and polysaccharides, including alginate and laminarin, among many others (Becker et al., 2020; Zhang et al., 2024). Several of the taxa we isolated are capable of using alginate, including *Algibacter* (Sun et al., 2016), *Cobetia* (Moriya et al., 2020), *Marinomonas* and *Psychromonas* (Thomas et al., 2021). Our WGS data indicated that *Vibrio*, *Olleya* and *Rhodoglobus* also have an alginate lyase enzyme suggesting that alginate use may be widespread in the seaweed microbiome. Laminarin, a dominant polysaccharide in brown algae (Hurd et al., 2014), is metabolised by β‐glucanase and β‐glucosidase enzymes and these were present in most of our isolated strains (Table S3). The two *Flavobacteriia* genomes we isolated had polysaccharide utilisation loci, *SusC* and *SusD*; these genes are also found in other *Bacteroidota* in the human gut (Grondin et al., 2017; Rakoff‐Nahoum et al., 2016), and are likely important across a wide variety of carbohydrate‐rich environments, including kelp. The inclusion of liquid extracted from kelp in our agar media resulted in two additional *Flavobacteriia* strains from the genus *Lishizhenia* and *Flavobacterium*; these taxa too may rely on kelp polysaccharides and highlight the role that host composition may play in the discovery and isolation of microbes. The importance of polysaccharides for ocean microbes may shape the genome, as suggested by a *Winogradskyella* strain that was isolated from an algal bloom and shown to have a streamlined genome for utilising laminarin as a sole polysaccharide (Alejandre‐Colomo et al., 2021).

Iron can be concentrated in the tissues of kelp (Miller et al., 2016) and could enhance enzymatic function in bacteria. For example, Ton B‐dependent starch‐binding outer membrane proteins in the *Flavobacteria* can transport iron and B vitamins (Noinaj et al., 2010). Further, iron could be an important currency for host–microbe interactions and *fhu* genes were present in 12 of the 16 isolates. Iron is the hypothesised ‘cost’ that drives genome loss and the *katG* dependency cited above for *Prochlorococcus* (Morris et al., 2012), emphasising its possible importance in microbial interactions.

### Bacterial metabolisms that may enhance host fitness

Microbial metabolisms may make DIN more available to the host via two metabolisms. First, Dissimilatory nitrate reduction recycles ammonium (DNRA), the most reduced form of nitrogen and an energetically less expensive form of nitrogen in seawater (Hurd et al., 2014). Complete metabolic modules for DNRA were present in *P. arctica* and *Vibrio* (Figure 2), and this ammonium source could be important for supporting the growth of seaweed hosts (Pritchard et al., 2015), including *Nereocystis*.

Our *Nereocystis*‐associated bacterial isolates were most successful on amino acids produced in high quantities on kelp; in turn, they provided the highest concentration of ammonium to the surrounding water. Specifically, most bacterial isolates grew best on asparagine and glutamine, the amino acids with two nitrogen atoms that are demonstrated to have the highest concentrations in seawater (Figure 3, Siezen & Mague, 1978) and seaweed (Wells et al., 2017), and also the amino acids with two nitrogen atoms. If ammonium is made available to the seaweed host by amino acid ammonification, a metabolism that was ubiquitous in our isolates, then nitrogen limitation to primary production in coastal marine systems could be alleviated in some instances (Galloway et al., 2008). Despite the ubiquity of amino acids in ocean water, the use of amino acids by indigenous bacteria is poorly described. Amino acids are a significant component of the DON release by eukaryotes (Myklestad et al., 1989; Smith, 1988; Tupas & Koike, 1990), and could be a valuable currency in host–microbe interactions. Whether this match between the highest isolate growth rates and the greatest availability of an amino acid is matched in other systems is unknown, but it is worthy of future study to determine how local environments shape microbial metabolism.

The association between ammonifying amino acids and seaweeds may be widespread in macrophyte‐microbiome systems. For instance, the functional capacity for ammonification was enriched in the giant kelp *Macrocystis* compared with surrounding seawater (Minich et al., 2018), as well as in cultures with the green alga *Ulva* (Shpigel et al., 2019) and ammonification was enhanced over tropical seagrass systems (Smith, 1988). We acknowledge that millimolar amino acid concentrations were used in our study, while the few studies that quantify amino acids in the coastal areas where these bacteria were cultured suggest seawater amino acid concentrations of 1 μM (Kuznetsova et al., 2004; Lee & Bada, 1977). Another measurement of DON concentration in the coastal northeast Pacific and in the vicinity of the origin of these isolates exceeds 10 μM (Berman & Bronk, 2003); amino acids could perhaps exceed 1 μM. While the millimolar concentrations of amino acids that we provided the bacterial isolates might be high relative to what is in seawater, there may be hotspots of amino acid production. For example, animal (Maas et al. 2020; Nagata & Kirchman, 1991) and phytoplankton excretion (Myklestad et al., 1989) of dissolved free amino acids generates high local concentrations of amino acids; the host *Nereocystis* may also, though this remains untested.

B vitamins are typically not produced by eukaryotic species suggesting that vitamin B production in our bacterial isolates is potentially essential to seaweed development (Brawley et al., 2017; Helliwell, 2017; Helliwell et al., 2011). Vitamins B_1_, B_6_ and B_7_ were present in many isolates, while vitamin B_12_ synthesis was restricted to *Vibrio*, *Psychromonas* and *Marinomonas*; neither the *Flavobacteriia* nor the *Actinomycetia* taxon showed evidence of vitamin B production and may themselves be auxotrophic for B vitamins (Gómez‐Consarnau et al., 2019).

### Other metabolisms that aid host‐associated bacteria

Inert or living surfaces in the coastal ocean can be areas of high bacterial densities and selection for protective metabolisms may result. The two strains of *Flavobacteriia* here (phylum *Bacteroidota*) have Type IX secretion systems, which are typically used as a means of movement, as a weapon, or as both (Lasica et al., 2017). We note that the *Flavobacteria* here did not have MACPF found in others, a membrane attack protein (perforin) that may serve in antagonistic interactions among bacteria and have been found in human gut *Bacteroides* (Chatzidaki‐Livanis et al., 2014).

Bacteria in association with seaweeds have also been implicated in morphological changes. We isolated strains of the genus *Marinomonas*, other strains of which control the morphology of its green seaweed host, *Ulva* (Singh et al., 2011). While we have no evidence yet for the same effect of *Marinomonas* on the kelp host we studied here, other studies have shown the importance of bacteria in the phenotype of seaweed, where development is thwarted in the absence of key taxa (Croft et al., 2005; Ghaderiardakani et al., 2017).

Finally, microbes on the surface of kelp may be detrimental to kelp growth. We found evidence of the agarase enzyme in the isolate *P. undina* and observed agarolytic activity on our agar plates. The species epithet of ‘*agarivorans*’ and ‘*carrageenavora*’ within *Pseudoalteromonas* and published accounts, suggest that these taxa may also have related enzymes for consuming algal polysaccharides (Gobet et al., 2018; Romanenko et al., 2003). It may be that bacteria that use the surface of kelp are in a dynamic relationship that can segue into consumption by the host. We note that other strains within *Pseudoalteromonas* have also been shown to rescue the red seaweed *Dilsea* from a pathogenic bacterial taxon (Li et al., 2021) and still others have inhibitory effects on heterospecific bacteria (Thomas et al., 2008).

Many of the bacteria associated with the bull kelp *N. luetkeana* appear to have metabolisms that take advantage of the unique kelp surface and potentially aid the host. Vitamin production and dissimilatory nitrate reduction could benefit the host, while host dissolved organic matter may benefit the microbes. Several bacterial genera that we isolated are found in other hosts, including humans. Mammalian oral and gut systems also represent environments with flow and carbon‐rich compounds, suggesting that similar selective pressures operate across hosts to determine the microbial community.

Marine species are increasingly recognised to host microbes that have fitness effects for the host, including corals (Zaneveld et al., 2017), cephalopods (Moriano‐Gutierrez et al., 2019), sponges (Zhang et al., 2015) and foundational macrophytes, such as seagrass (Mohr et al., 2021; Tarquinio et al., 2018) and seaweeds (Li et al., 2022). Our capacity to understand the multiple ways in which microbes may have beneficial or detrimental effects for critical marine species relies on the development of experimental systems of inquiry for host–microbe interactions. The methods, isolates and analyses we report here are a first step to identify the role of microbes for the foundational canopy kelp *N. luetkeana* in its indigenous environment. Our work is also fundamental to developing this critical species as a model system for understanding temperate kelp fitness under global change, its use for blue carbon sequestration and its potential in restoration and aquaculture.

## AUTHOR CONTRIBUTIONS

**Isaac T. Younker:** Conceptualization (equal); data curation (equal); formal analysis (equal); investigation (equal); methodology (equal); validation (equal); visualization (equal); writing – original draft (equal); writing – review and editing (equal). **Nichos Molnar:** Investigation (equal); validation (equal); writing – review and editing (equal). **Kaylie Scorza:** Investigation (equal); validation (equal); writing – review and editing (equal). **Roo Weed:** Investigation (equal); methodology (equal); validation (equal); writing – review and editing (equal). **Samuel H. Light:** Conceptualization (equal); funding acquisition (equal); methodology (equal); resources (equal); writing – original draft (equal); writing – review and editing (equal). **Catherine A. Pfister:** Conceptualization (equal); data curation (equal); formal analysis (equal); funding acquisition (equal); investigation (equal); methodology (equal); resources (equal); visualization (equal); writing – original draft (equal); writing – review and editing (equal).

## CONFLICT OF INTEREST STATEMENT

The authors declare no conflict of interest.

## Supporting information


**Data S1.** Supporting Information.

**Data S2.** Supporting Information.

## Data Availability

Supporting Information tables have all data used in analyses. All sequence files submitted to NCBI under project number PRJNA1076209. Reviewers can access these sequence files on a shared drive: Younker et al. Fasta files.
