# Supplementary material for: Bacteria on the foundational kelp in kelp forest ecosystems: Insights from culturing, whole genome sequencing and metabolic assays
Source: Environ Microbiol Rep. 2024 May 22;16(3):e13270. doi: 10.1111/1758-2229.13270 (PMC11112141; doi:10.1111/1758-2229.13270)
Supplement: Supplementary file 2 — Data S2. Supporting Information. [file EMI4-16-e13270-s001.docx]

**Supporting Information for** “*Bacteria on the foundational kelp in kelp forest ecosystems: insights from culturing, whole genome sequencing, and metabolic assays*”

**Supplemental Tables (attached as a separate Excel file)**

**Table S1.** Enzyme Commission numbers within each genome that would indicate enzymes that act on CH-NH2 bonds (EC:1.4.*), enzymes that act on carbon-nitrogen bonds other than peptides (EC:3.5.*) or ammonium lyases (ED:4.3.1*), where * indicates any subset of these classifications.

**Table S2.** The ASVs in each sample from the 3 sites, based on 16S rRNA amplicon sequencing. The taxonomic assignment to Genus and the sequence are given. The taxa are shown by Order in Figure 1c, and the Phylum assignment for the top 100 ASVs by count are shown in Figure 1d.

**Table S3.** Isolates and their genomic and metabolic features, displayed in Figure 2 and Figure S3. The metabolisms shaded in blue are those shown in Figure 2.

**Table S4.** Module completion scores (from 0.0 to 1.0) for all 16 Bacterial isolates based on whole genome sequencing. Module completion was estimated with anvi'o (https://anvio.org/) using anvi-estimate-metabolism (https://merenlab.org/m/anvi-estimate-metabolism).

**Table S5.** Kegg hits for every KO number and all 16 Bacterial isolates based on whole genome sequencing. Kegg matches were estimated with anvi'o (https://anvio.org/) using anvi-run-kegg-kofams and default parameters in anvi’o of a default threshold (1e-05) and a bitscore of 0.5.

**Table S6.** Statistical results.

**Supplemental Figures**


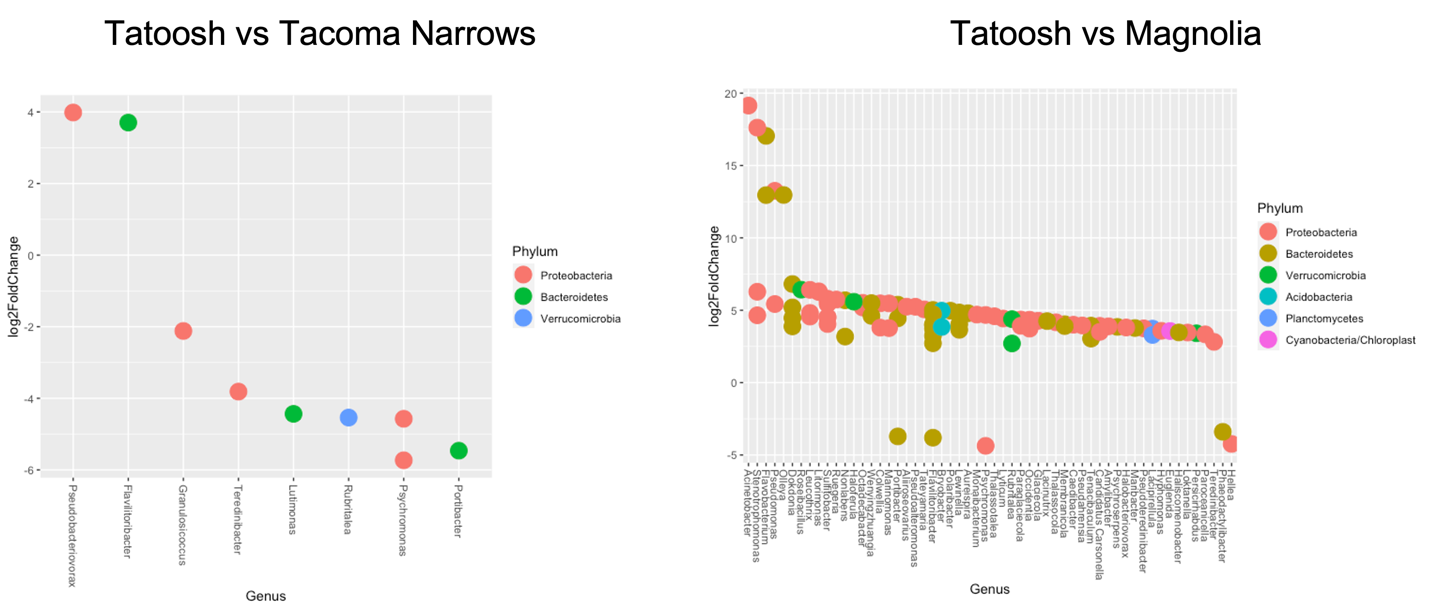


Figure S1. The differential abundance of ASVs at Tatoosh vs Tacoma Narrows and Tatoosh versus Magnolia that significantly differed in relative abundance (Deseq2 in R) and based on 16S data from Table S2.


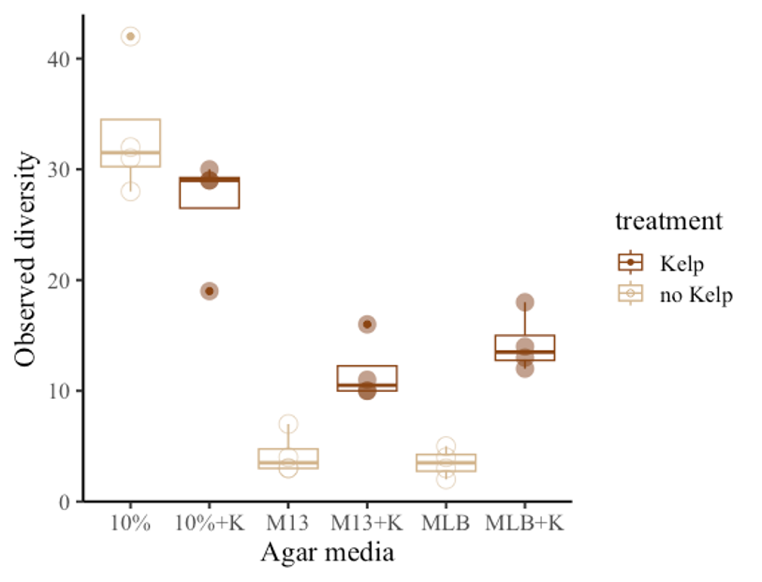


Figure S2. The observed ASV diversity when kelp liquid was added or left out of 3 media types. The 10% MLB agar did not show an increase in taxa discovery with kelp liquid, but there were

3 bacterial strains that were unique to the addition kelp extract to agar media, including two Bacteroidota in the Flavobacteriia (*Lishizhenia*, *Flavobacterium*) and a Gammaproteobacteria in the Halomonadaceae (*Cobetia*).


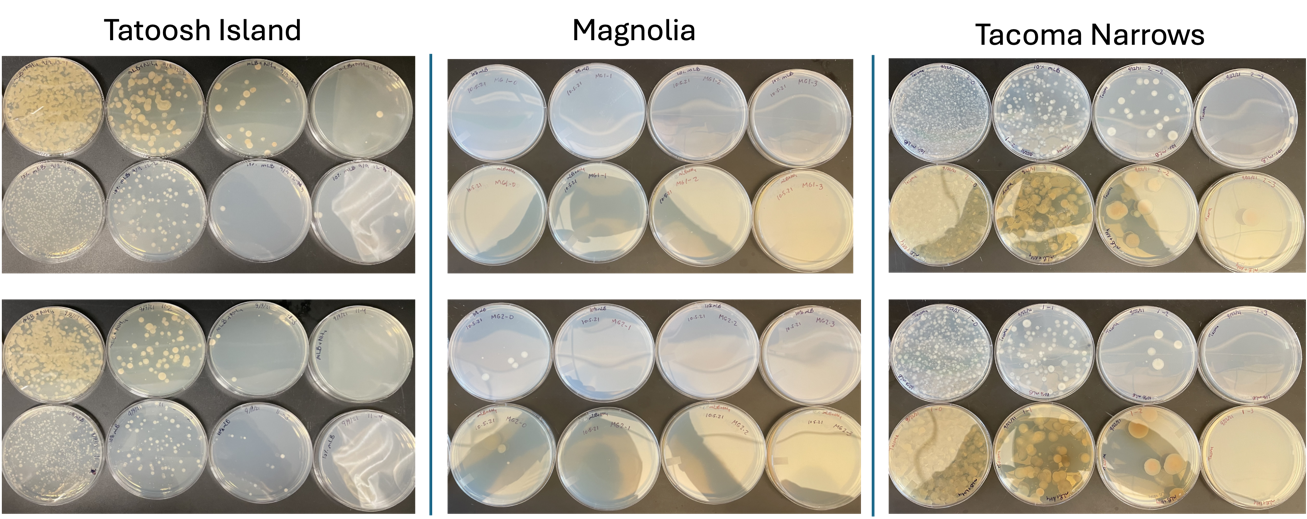


Figure S3. Pictures of the cultures from directly plating kelp homogenate and used in isolating bacteria from the bull kelp *Nereocystis luetkeana* from 3 sites. The plates show dilutions from left to right for 2 blades at each site plated on MLB +NH_4_ and 10%MLB. Cell densities were always higher at Tatoosh Island and numbered 10^3^-10^5^ CFU/mL, while Tacoma Narrows was 10^3^-10^4^ CFU/mL, and Magnolia was 0-10 CFU/mL.


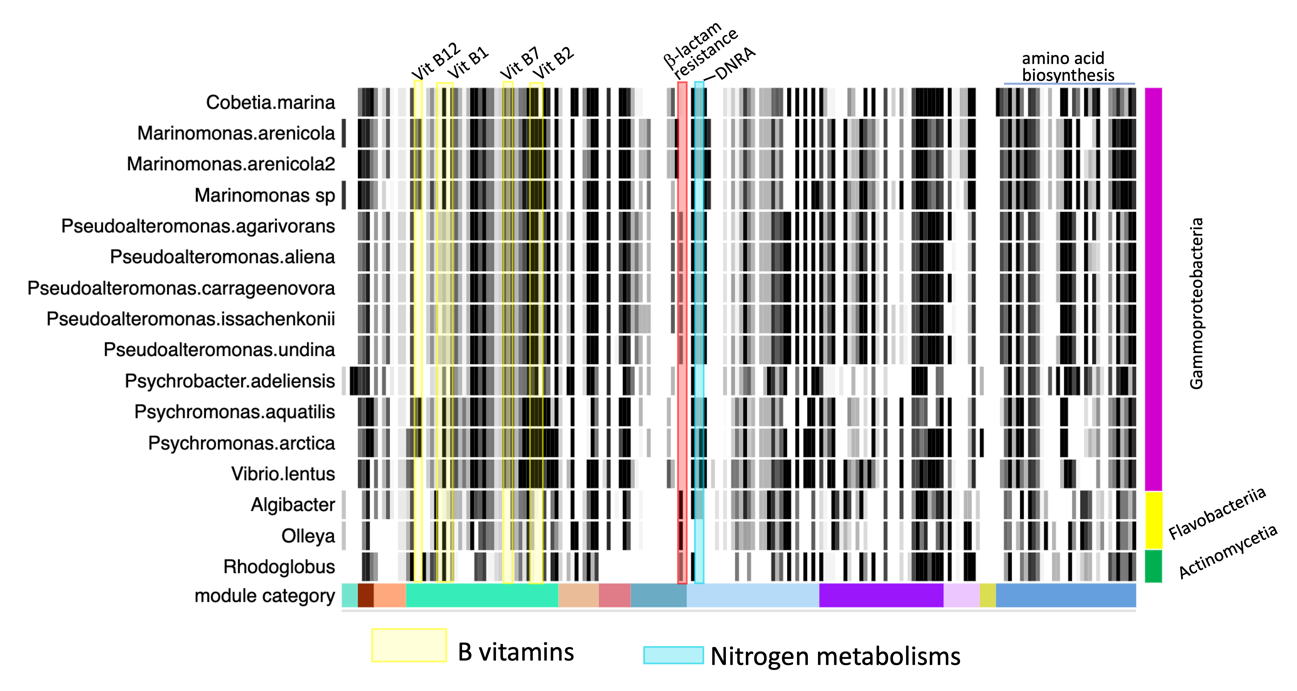


Figure S4. The degree of completion of metabolic modules that differed among the 16 bacterial isolates displayed as a heatmap. Each column represents a metabolic module and is shaded from white to black, where white is a module with less than 30% completion. We highlight B vitamins, antibiotic activity, dissimilatory nitrate reduction to ammonium (DNRA) and a broad range of amino acid biosynthesis. The full list of metabolic modules and their completion scores are in Table S4.
